# Assessing the Impact of Training on Healthcare Providers' Adherence to Infection Control Measures in Hemodialysis Services

**DOI:** 10.7759/cureus.42978

**Published:** 2023-08-05

**Authors:** Sukhbir Singh, Hemchandra Pandey, Hari K Aggarwal, Shekhar Pal

**Affiliations:** 1 Hospital Administration, Pandit Bhagwat Dayal Sharma Post Graduate Institute of Medical Sciences, Rohtak, IND; 2 Administration, Hemwati Nandan Bahuguna Medical Education University, Dehradun, IND; 3 General Medicine, Pandit Bhagwat Dayal Sharma Post Graduate Institute of Medical Sciences, Rohtak, IND; 4 Microbiology, Government Doon Medical College, Dehradun, IND

**Keywords:** central venous catheters (cvcs), catheter-related bloodstream infections (crbsis), hemodialysis services, health care providers (hcps), practice, structured training program

## Abstract

Background and objective

Developing and implementing nursing interventions to educate nurses on infection control procedures in hemodialysis units is of utmost importance and offers significant benefits in enhancing the quality of care. This study aimed to assess the impact of training on nursing professionals’ practices of hospital infection control measures in hemodialysis services. The research also intended to explore the potential association between these practices and various sociodemographic variables.

Materials and methods

This was a single-group, pre- and post-interventional study carried out in Haryana State, India. A pretested questionnaire consisting of 29 statements, the responses of which were measured on a 5-point Likert scale, was used as the study tool. Descriptive and statistical tests like paired-t-test were used to analyze the data.

Results

The practices section of the questionnaire comprised 29 statements, the responses to which were measured on a five-point Likert scale. The scoring ranged from 5 ("strongly agree", i.e., positive practice) to 1 ("strongly disagree", i.e., negative practice). The maximum achievable score was 145 and the minimum achievable score was 9. The pre-test group (i.e., before training intervention) had a mean practice score of 115.0945 [standard deviation (SD)=9.34, standard error of the mean (SE)=0.66]. However, the post-test group (i.e. after training intervention) had a mean score of 135.26 (SD=8.34, SE=0.59). The study found that structured training significantly increased the mean practice score (t=-33.70, p=0.001). In addition, the study also highlighted the significant association of mean practice scores with various demographic variables among the pre-test and post-test groups. The improvement in mean practice scores among the post-test group after the structured training program reveals that such interventions will ultimately lead to a decrease in central line-associated bloodstream infections (CLABSIs) among hemodialysis patients.

Conclusions

Our findings showed that the educational intervention led to significant improvements in the practices of the participants.

## Introduction

Patients undergoing hemodialysis are at an increased risk of infection, which poses a significant clinical challenge due to its association with elevated morbidity and mortality [1]. There is scarce data regarding the prevalence and impact of comorbidities and complications associated with kidney failure. Heart disease and infections account for approximately two-thirds of total mortality [2]. One of the primary infections experienced by hemodialysis patients is associated with dialysis catheters [3]. India has a higher burden of kidney failure-related deaths compared to other low- and middle-income economies with comparable sociodemographic indicators, indicating potential for improvement in death rates [4]. Central venous catheters (CVC) have come to play an essential role in the care of patients undergoing hemodialysis, but they can lead to catheter-related bloodstream infections (CRBSIs) and some patients could potentially succumb to sepsis and hyperinflammatory syndromes. Utilizing CVCs is associated with various complications that elevate patient morbidity and death rates, as well as increased treatment costs and prolonged hospital stays [5].

Advancements in technology have led to an escalation in the intricacy of hemodialysis treatment, exposing patients to an elevated risk of infection from various causes associated with chronic illnesses [6]. Infection is a prevalent factor leading to hospitalization among individuals undergoing hemodialysis treatment and has emerged as the second most important reason for mortality in this patient population. Proper hygiene practices among staff members are crucial to preventing infection [7]. As primary caregivers, they occupy a distinctive position to exert influence on infection prevention practices and patient treatment outcomes [8]. Developing and implementing nursing interventions to educate nurses on infection control practices in hemodialysis units is of paramount importance and offers significant benefits in enhancing the quality of care [9]. A recent study has reported that an upskilling program considerably improved nurses' understanding of caring for patients with CVCs, positively impacting patient outcomes [10]. Ongoing research and widespread sharing of evidence are crucial in enhancing nurses' understanding of CVC maintenance care, with the ultimate goal of reducing catheter-related bloodstream infections (CRBSIs) and ensuring patient safety [11].

Given this context, we conducted this study at a reputed tertiary care hospital in northern India to assess the impact of training on nursing professionals’ practices of hospital infection control measures in hemodialysis services. We also aimed to explore the potential association between these practices and various sociodemographic variables.

## Materials and methods

Study setting and design

The research was carried out at the Pandit Bhagwat Dayal Sharma Post Graduate Institute of Medical Sciences in Rohtak, Haryana. This institution is a prominent center for academic pursuits, research, and advanced medical care in the state of Haryana. The study involved pre- and post-single-group interventions (n=208). All nursing professionals posted in study areas were enrolled in the study. The study setting encompassed various hospital areas including the Dialysis Unit, Nephrology Ward, Urology Ward, and Medicine Wards. Additionally, the study also included ICUs situated within the New OT Cum ICU Complex, the Emergency Department, the Dhanwantri Apex Trauma Centre, and the Day Care ICU. The study did not involve healthcare providers such as faculty members, resident doctors, paramedical staff, or other support staff who were assigned to areas under study. Additionally, nursing professionals stationed outside the designated study areas were not included in the research. Nursing professionals who were eligible for the study but did not provide informed consent were also excluded. The participants were selected by convenience sampling technique. Participation was on a voluntary basis. Three participants opted out of the study. The current study received approval from the Institute Ethics Committee (letter no. BREC/21/28, dated 03/05/2021). Informed consent was obtained from each participant before administering the pre- and post-tests.

Study tool

A questionnaire was created to assess practices related to hospital infection control measures in hemodialysis services. The questionnaire was developed based on guidelines from the Government of India and the Indian Society of Nephrology, as well as the CDC and WHO guidelines and a literature review. The questionnaire had two parts: the first part gathered sociodemographic information about participants. The second part dealt with the assessment of various practices of participants with regard to the study topic and comprised 29 statements. The responses to these statements were measured on a Likert scale of five points for evaluating the practices of the participants. The scores on the Likert scale aligned with the following responses: strongly agree, agree, neither agree nor disagree, disagree, and strongly disagree. The validity of the questionnaire was tested by applying Cronbach’s alpha test for reliability analysis for all groups, i.e. pre- and post-test groups. Our scale's consistency was found to be acceptable with a Cronbach's alpha over 0.70. The study tool, i.e., questionnaire, covered the following domains related to infection control practices in hemodialysis services: (a) hand hygiene; (b) glove use and other personal protection; (c) environmental concerns, including consumables and equipment; (d) cleaning of chairs, beds, and dialysis machines; (e) disinfection of hemodialysis machines; (f) proper handling of dialysates; (g) appropriate handling of medications. (h) blood-borne virus screening and management and multi-resistant organisms (MROs); (i) biomedical waste management; (j) management of blood spills, etc. (Table 1).

**Table 1:** Details regarding the study tool IEC: Information, Education, and Communication; PPE: personal protective equipment

Statement regarding healthcare providers' practices	Total number of questions/statements in the questionnaire
Display of IEC material regarding infection control practices reminds healthcare providers to perform hand hygiene	01
Healthcare providers' practices regarding hand hygiene	02
Influence of hospital's infection prevention team on healthcare providers' hand hygiene practices	01
Impact of appropriate hand hygiene practices by healthcare providers on medical costs associated with nosocomial infections and on patient morbidity and mortality	01
Healthcare providers' practices regarding wearing gloves while providing hemodialysis services	02
The practice of using spectacles and contact lenses by healthcare providers	01
The practice regarding plastic aprons for preventing clothing infection from blood, bodily fluids, and other potentially contagious materials	01
Correct practice of donning and doffing of PPE	01
Correct practice of disinfection and/or disposal of PPE	01
The practice of using a patient's consumables solely for that patient and avoiding returning them to a common clean area or using them on other patients	01
The practice that blood tubing of the hemodialysis machine should be draped or clipped to waste containers	01
Practice during the priming of dialyzers, to make use of the waste containers that are attached	01
The practice of placing items such as dialyzer caps and medication vials on top of hemodialysis machines is common	01
Practice related to disinfection, and cleaning of the machine after each patient	01
Practice related to frequent bleaching of the machine	01
The practice of immediately capping unused dialysate and washing the exterior with water and detergent	01
The practice of recording the date and time of opening the bottle with a permanent pen	01
The practice of handling and storing medications or clean supplies and the use of multi-dose vials for the same patient	02
The practice of washing trays in between use to administer drugs to specific patients	01
The practice of changing gowns and gloves and hand washing	01
If isolation facilities are not available, practice of isolating positive patients from susceptible patients, and dialysis of these patients using dedicated machines	01
The practice of applying topical antibacterial ointments to the exit site in central venous catheter-dialysis patients	01
Practice regarding manually recapping needles	01
The practice of using appropriate color-coded bins for the disposal of different categories of biomedical waste	01
The practice of segregating infectious waste and non-infectious waste	01
Proper blood spill management practices	01

Tool administration

The researchers reached out to the participants and invited them in groups to take a pre-intervention (pre-test) questionnaire. This questionnaire was used to measure the participants' existing practices on the topic of the research. The respondents were asked for their written consent before handing out the questionnaire, which they then returned after completing it. After the pre-test, the participants were provided with a structured training program. This program was developed after researching relevant literature [12-14] and guidelines, and consulting with subject matter experts. The curriculum was in English and included specialized lectures delivered via audiovisual aids, as well as hands-on training sessions for the participants. Once the training was complete, the participants were given the same questionnaire to assess the effect of the education and training intervention on their practices. Their written consent was obtained before administering the post-test as well. The pre-test, training, and post-test assessments were conducted in batches and a similar procedure was followed for all participants. This study was part of a research project by the first investigator. The training of the study participants was conducted by the investigator, who is a professor and subject expert. On average, 40 participants were trained in one batch.

Data analysis

The practices section of the questionnaire had 29 statements, the responses of which involved scoring on a five-point Likert scale. The responses available were as follows: strongly agree, agree, neither agree nor disagree, disagree, and strongly disagree. The scoring ranged from 5 (strongly agree, i.e., positive practice) to 1 (strongly disagree, i.e., negative practice). The collected data were entered into a Microsoft Excel sheet. The entered data was assessed for cosmetic and logical errors. A cleaned master data was then fed to IBM SPSS Statistics version 23.0 (IBM Corp., Armonk, NY) for further analysis. Frequency analysis and descriptive statistics were performed to find the distribution of quantitative and qualitative variables respectively. Paired sample t-test was applied to see if there was any noteworthy variation between pre- and post-test evaluation on the KAP of selected participants.

## Results

The study included 208 participants, the majority of whom were married females (86%) and residents in urban areas (81%). Most of the participants held a General Nursing & Midwifery diploma (52%) and were nursing officers (88%) with 0-5 years of professional experience (39%). The pre-test group had a mean practice score of 115.0945 [standard deviation (SD)=9.32502, standard error of the mean (SE)=0.65774] while the post-test group had a mean of 135.2637 (SD=8.33757, SE=0.58809). The study found that structured training significantly increased the mean practice score (t=-33.704, p=0.001) (Table 2).

**Table 2:** Mean practice scores among pre- and post-test groups of participants

Parameter (practice)	Mean	N	Std. deviation	Std. error of the mean	t statistic, p-value
Pre-test group	115.0945	201	9.32502	0.65774	-33.704, 0.001
Post-test group	135.2637	201	8.33757	0.58809	

Analysis showed that the post-test group had significantly higher practice scores than the pre-test group across all age groups (t=-21.816, df=77, p<0.001). The post-test group also had considerably high scores than the pre-test group among both married and unmarried individuals (t=-30.416, df=168, p<0.001 and t=-14.489, df=31, p<0.001, respectively) and for both males and females (t=-13.620, df=28, p=0.001 and t=-30.840, df=171, p=0.001, respectively). A statistically noteworthy increase in mean practice score for the post-test group was found across all educational qualifications compared to the pre-test group: GNM, BSc Nursing, and MSc Nursing (t-values: -25.100, -21.401, and -7.715, respectively). This was true for residential locations also, with the mean practice score of the post-test group higher than that of the pre-test group among nurses from both rural and urban areas (t=-17.401, df=38, p=0.001 and t=-29.634, df=161, p=0.001, respectively). The average practice scores of the post-test group were appreciably higher than those of the pre-test group for both Nursing Officers and Senior Nursing Officers (t=-31.936, df=176, p=0.001, and t=-10.932, df=23, p=0.001, respectively). The post-test group also had a much higher mean practice score for all experience levels: 0-5 years, 6-10 years, 11-15 years, and more than 15 years (t=-19.716, df=77, p=0.001; t=-18.005, df=48, p=0.001; t=-15.511, df=35, p=0.001; and t=-13.888, df=37, p=0.001, respectively). This was the case with posting locations as well, with the average level of practice in the post-test group significantly greater than that of the pre-test group in both Ward and ICUs (t=-23.825, df=92, p=0.001 and t=-24.141, df=107, p=0.001, respectively) (Table 3).

**Table 3:** Mean practice scores in pre- and post-test groups with regard to various sociodemographic parameters GNM: General Nursing & Midwifery

Parameter		Mean	N	Std. deviation	Std. error of the mean	t	df	P-value
Age group, years								
21-30	Pre-test	116.7564	78	8.39788	0.95087	-21.816	77	0.001
	Post-test	135.4359	78	8.43091	0.95461			
31-40	Pre-test	114.8205	78	9.83202	1.11326	-23.375	77	0.001
	Post-test	135.3205	78	7.73337	0.87563			
41-50	Pre-test	112.0000	19	10.65103	2.44351	-9.052	18	0.001
	Post-test	132.2632	19	9.64274	2.21220			
>50	Pre-test	113.1923	26	8.89503	1.74446	-10.665	25	0.001
	Post-test	136.7692	26	8.76040	1.71806			
Marital status								
Married	Pre-test	114.9053	169	9.59678	0.73821	-30.416	168	0.001
	Post-test	135.0000	169	8.49159	0.65320			
Unmarried	Pre-test	116.0938	32	7.78899	1.37691	-14.489	31	0.001
	Post-test	136.6563	32	7.43839	1.31493			
Gender								
Male	Pre-test	119.6207	29	8.10826	1.50567	-13.620	28	0.001
	Post-test	139.1724	29	7.03597	1.30655			
Female	Pre-test	114.3314	172	9.32083	0.71071	-30.840	171	0.001
	Post-test	134.6047	172	8.37677	0.63872			
Educational qualification								
GNM	Pre-test	114.1524	105	9.48053	0.92520	-25.100	104	0.001
	Post-test	134.3619	105	8.52386	0.83184			
BSc Nursing	Pre-test	116.2889	90	8.95337	0.94377	-21.401	89	0.001
	Post-test	136.1000	90	8.21488	0.86592			
MSc Nursing	Pre-test	113.6667	6	11.62182	4.74459	-7.715	5	0.001
	Post-test	138.5000	6	5.08920	2.07766			
Place of residence								
Rural	Pre-test	116.7179	39	9.42547	1.50928	-17.401	38	0.001
	Post-test	135.1538	39	8.47454	1.35701			
Urban	Pre-test	114.7037	162	9.28760	0.72970	-29.634	161	0.001
	Post-test	135.2901	162	8.33065	0.65452			
Designation								
Nursing Officer	Pre-test	115.1921	177	9.46929	0.71176	-31.936	176	.001
	Post-test	135.1695	177	8.24859	0.62000			
Senior Nursing Officer	Pre-test	114.3750	24	8.32917	1.70018	-10.932	23	0.001
	Post-test	135.9583	24	9.12464	1.86256			
Experience, years								
0-5	Pre-test	115.5513	78	9.73499	1.10227	-19.716	77	0.001
	Post-test	134.5128	78	8.95841	1.01434			
6-10	Pre-test	116.1429	49	7.96607	1.13801	-18.005	48	0.001
	Post-test	137.0816	49	6.75412	0.96487			
11-15	Pre-test	112.5833	36	11.20555	1.86759	-15.511	35	0.001
	Post-test	133.8333	36	8.48360	1.41393			
>15	Pre-test	115.1842	38	7.97921	1.29440	-13.888	37	0.001
	Post-test	135.8158	38	8.57985	1.39184			
Place of posting								
Ward	Pre-test	114.9462	93	9.69689	1.00552	-23.825	92	0.001
	Post-test	136.3226	93	7.29973	0.75695			
ICUs	Pre-test	115.2222	108	9.03610	0.86950	-24.141	107	0.001
	Post-test	134.3519	108	9.07221	0.87297			

The study instrument consisted of several statements that covered different practices related to the topic of research. It was found that during the pre-intervention phase itself, a significant proportion of participants (43%) strongly agreed that the infection prevention team at the hospital has a positive influence on the hand hygiene habits of healthcare providers. However, there was still an improvement in this figure after the educational intervention, with 68% of respondents strongly agreeing that this practice statement is accurate. Similarly, before the intervention, only 39% of the respondents strongly agreed that wearing gloves while engaging in any activity requiring cleaning is the best practice. However, after the training, a massive 71% of the participants strongly thought that this practice statement was accurate. Furthermore, the research highlighted significant improvements in the participants' practices with respect to the use of eyeglasses and contact lenses for eye protection, the use of plastic aprons for preventing clothing infection from blood, bodily fluids, and other potentially contagious materials, the correct practice of donning and doffing of personal protective equipment (PPE) kits, the proper practice of disinfecting and disposing of used PPE kits, and practices related to disinfection. Similarly, significant improvement was also reported in a few other practices, such as marking the day and time of bottle opening with a permanent pen, utilizing appropriate color-coded bins for the disposal of different types of biomedical waste, etc. These improved practices will ensure that healthcare providers can carry out their duties safely and efficiently, thereby delivering high-quality patient care leading to a decrease in CLABSI infection among hemodialysis patients.

## Discussion

Our findings revealed the positive impact of training/educational intervention on the infection control practices of the healthcare providers related to hemodialysis services. Out of the total identified Nursing & Senior Nursing Officers, 96.63% participated in this study. Males accounted for 16.86% of the study participants, which is in line with previous research carried out by Deshmukh and Shinde (16.67%) [15] and slightly lower than the findings of the study by Bianco et al. (33.8%) [16]. This finding may be explained by cultural factors, which encourage more females to enroll in the nursing profession. Hence, female participants constituted the predominant demographic of the study, accounting for 85% of the entire sample, and in the age range of 21-40 years. It is important to point out that before the intervention, the cohort had an average practice score of 79.37% of the highest possible score. However, after the intervention, the cohort's average practice score increased to 93.29% of the total achievable score. Hence, the research findings indicate that implementing educational/training interventions substantially improved the infection control practices of healthcare professionals. Our findings revealed that after educational intervention, the improvement in practice score was higher among the unmarried, male participants, urban participants, and participants with a nuclear family background, senior nursing officers, participants posted in wards, when compared to their counterparts who were married, females, residing in rural areas, from a joint family background, nursing officers, and participants posted in ICUs.

The higher level of improvement among the unmarried and male participants may be attributed to the fact that their baseline practice scores were already better than their married and female counterparts. However, the pattern of improvement in a few of the subgroups was not linear and is not easy to explain and it may be ascribed to the disparity in the size of the sample. On age group-wise analysis, it was found that the post-intervention improvement in practice score was the highest among the participants in the age group of more than 50 years. However, there was no linear pattern among the different age groups. On the other hand, when analyzing improvement based on educational qualifications, the improvement in practice score was the highest in the postgraduate participants and the pattern of improvement was linear, i.e., the maximum improvement was observed among the postgraduates followed by the graduates and diploma holders. This trend may be attributed to the fact that the participants with a postgraduate degree had been exposed more to the study topic through their advanced study curriculum. Regarding analysis based on experience, it was found that the enhancement in practice score was the highest among participants with 6-10 years of experience, which may be explained by the fact that healthcare providers are more practically involved with these patients in the early-middle part of their careers. The current investigation results are corroborated by a prior study completed in 2016 by Metwally et al., which showed that nurses with 5-10 years of experience or less had a higher average percentage practice score compared to those with over 10 years of experience [17]. This study is remarkable for its emphasis on infection control measures associated with hemodialysis services. The educational intervention among healthcare providers was effective, as demonstrated by a substantial change in practice scores after the intervention compared to pre-intervention. The findings of this study may contribute to enhancing infection control strategies and minimizing the occurrence of CLABSI in hemodialysis patients.

This study has a few limitations. This was a single-center study, and the participants were chosen from a particular group of healthcare professionals. Therefore, it is uncertain if the conclusions can be generalized to all types of healthcare professionals or hospitals.

## Conclusions

This study aimed to explore how training can impact healthcare providers' infection control practices in hemodialysis services. The results showed that the educational intervention led to significant improvements in the practices of healthcare providers. However, the study also highlighted areas where there is still room for improvement. While the training had a positive impact on healthcare providers' infection control practices, further research is needed to determine whether they routinely apply evidence-based best practices and retain the understanding gained from their training.
